# Effects of weight change on taste function; a systematic review

**DOI:** 10.1186/s12937-023-00850-z

**Published:** 2023-05-08

**Authors:** Mojdeh Fathi, Ahmad Zare Javid, Anahita Mansoori

**Affiliations:** 1 Department of Clinical Nutrition, School of Nutrition and Food Science, Isfahan University of Medical Sciences, Isfahan, Iran; 2 Department of Nutrition, School of Allied Medical Sciences, Ahvaz Jundishapur University of Medical Sciences, Ahvaz, Iran; 3 Nutrition and Metabolic Diseases Research Center, Ahvaz Jundishapur University of Medical Sciences, Ahvaz, Iran; 4 Nutrition and Metabolic Diseases Research Center, Clinical Sciences Research Institute, Ahvaz Jundishapur University of Medical Sciences, Ahvaz, Iran

**Keywords:** Taste perception, Taste sensitivity, Taste threshold, Taste preference, Weight loss, Weight gain

## Abstract

**Background:**

The aim of this review is to evaluate the relationship between weight status and taste perception and preference of sweet, salt, fat, bitter, and sour through reviewing observational and interventional studies with objective methods.

**Methods:**

A comprehensive literature search was performed in 6 online databases of PubMed, Scopus, Web of Science, Cochrane, Embase, and Google Scholar up to October 2021. The following keywords were used in the search strategy: (Taste OR "Taste Perception" OR "Taste Threshold" OR "Taste preference" OR "Taste sensitivity" OR "Taste changes") AND (weight OR "Weight gain" OR "weight loss" OR "weight change").

**Results:**

Most observational studies indicate that four taste sensitivities or perceptions (especially sweet and salt taste perception) are lower in subjects with overweight and obesity. The longitudinal studies reported that sweet and fat preference is increased along with weight gain in adults. It is concluded that taste perceptions are decreased in individuals with overweight and obesity, especially in men. Also, taste perception and preference change after weight loss but not significantly.

**Conclusion:**

It is suggested that the results of the interventional studies are not conclusive and need further studies with the same and standard design adjusting cofounding variables including genetic, gender, age and food condition of subjects.

**Supplementary Information:**

The online version contains supplementary material available at 10.1186/s12937-023-00850-z.

## Background

Obesity, with severe complications including hypertension, cardiovascular disease, type 2 diabetes and certain types of cancer, is an increasing global health threat in children and adults resulting to economic and social consequences. According to a predictive model, the prevalence of obesity will reach 48.9% by the year 2030 among US adults [1]. There are several factors including genetics, socioeconomic status, medical condition, gut microbiota and the gut-brain axis, lifestyle, and individual preferences involved in the pathogenesis of obesity [2]. Some of main reasons to choose a certain food to eat are availability, culture, cost, attitude, and taste [3].

Taste sensation, one of the five main senses, helps humans to determine five basic tastes of sweet, salty, bitter, sour, and umami- and recognize nutritious and harmful substances [4].The tastes of sweet, umami, and salty have encouraging effects on consumption of certain foods as sources of calories, proteins, and minerals; whereas the bitterness and sourness have the avoiding effects on eating toxic and/or spoiled foods [5]. Several studies suggest that fat has specific receptors in taste cells and is considered as a sixth taste modality [6, 7]. The effects of taste preference and perception on obesity and the vice versa are still unclear. It indicated that taste perception impacts on individual food preference and eating behavior and consequently on body weight [8]. However, Berthoud and Zheng suggest that the weight change and sense of taste is correlated and the weight loss can alter taste sensitivity. One potential mechanism involved in taste alteration linked to weight changes is leptin signaling, as it can modulate taste processing [9]. Moreover, some studies indicate that diet can modulate taste receptor expression [10]. However, current evidence from observational and interventional studies on modulation of the sense of taste (perception, intensity, sensitivity, threshold) by weight changes is conflicting. Although some studies reported a positive correlation between weight change and taste perception [11], there are some reports suggesting a negative correlation [12]. Some evidence showed no relation between sweet taste preference and obesity [13]. Such controversial findings may be due to different techniques used in studies to evaluate taste. Furthermore, the gender and age of subjects should be considered [14]. Therefore, apart from the effects of obesity on taste or vice versa, the taste stimulation would be important practically for food industries and restaurants where it is tried to attract customers through stimulating the taste. So, it is assumed that the prevalence of obesity will increase in the worldwide.

Overall, a systematic review is needed to summarize all available findings in this issue. Therefore, the current review was done to evaluate the relationship between taste and weight changes and discuss the evidence on the effects of weight changes on sense of taste (perception, intensity, sensitivity, threshold, preference) in children, adolescence and adults. It is suggested that a better understanding of the relationship between weight change and taste can provide an opinion in terms of a food choice and eating habits.

## Methods

### Search strategy and study selection

**Fig. 1:**
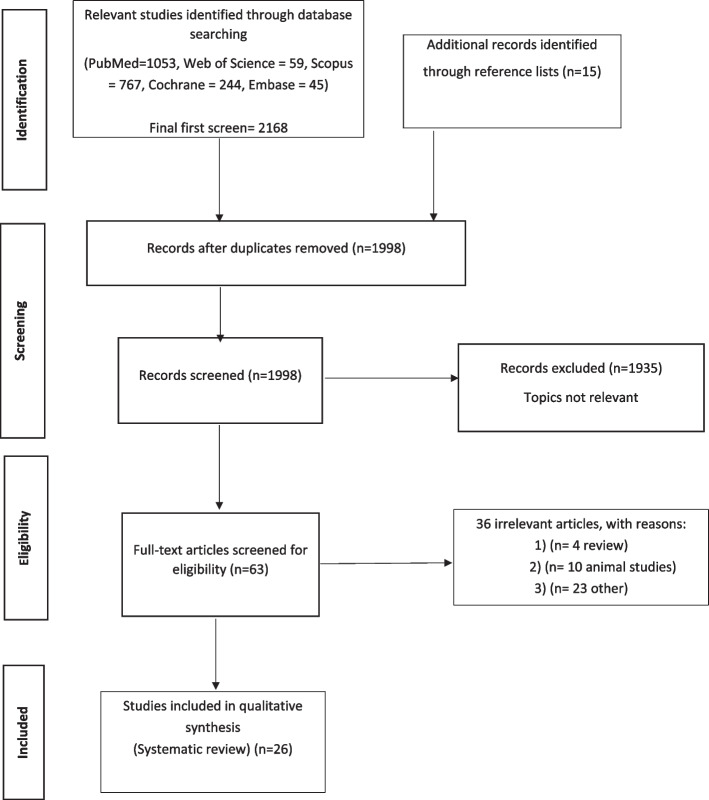
Study selection flow chart

This study was conducted based on Preferred Reporting Items for Systematic Reviews and Meta-Analysis (PRISMA) protocol. A comprehensive literature search was performed in 6 online databases of PubMed, Scopus, Web of Science, Cochrane, Embase, and Google Scholar up to February 2023. The following keywords were used in the search strategy: (Taste OR "Taste Perception" OR "Taste Threshold" OR "Taste preference" OR "Taste sensitivity" OR "Taste changes") AND (weight OR "Weight gain" OR "weight loss" OR "weight change"). In addition, the reference list of the relevant papers was checked to avoid missing any publication. All searched studies were included in the Endnote software for screening. Duplicate citations were removed afterward. The human studies which evaluate the effect of weight changes on taste (preference, threshold, sensitivity) in children, adolescence and adults were included. No restriction was made at the time of publications and language and type of study. All observational studies with cross-sectional, case–control, or cohort design and trial studies conducted on weight and taste status were included. All studies that were used a direct measurement scale such as using filter paper for assessment of taste perception and special foods assessment for assessment of taste preference were included. The exclusion criteria involved: studies in which the subjects suffered from any disease and used any prescription; studies with the interventions of weight loss using any drug; studies in which an overall food questionnaire was included in taste scale; unpublished studies, review articles, papers with abstract only, and presentations. The number of articles included and excluded in the study selection process is shown in the flowchart below (Fig. 1 Study selection flowchart).

### Data extraction

The data extraction out of the included articles were independently performed by two authors. The following information was extracted: the name of the first author, the publication year, study design, population characteristics (age, gender, and weight status), duration of the intervention (for interventional studies), and method of taste assessment.

### Quality assessment

The Cochrane quality assessment tool was used to assess the bias risk for the interventional eligible studies by two reviewers independently [15]. This tool contains six domains including random sequence generation, allocation concealment, reporting bias, performance bias, detection bias, and attrition bias. Each domain was given a score as “high risk” if the study comprised methodological defects affecting its findings, “low risk” if there was no defect for that domain, and “unclear risk” if the information was insufficient to determine the impact. The overall risk of bias for a randomized clinical trial (RCT) study was considered as low if all domains had “low risk” score; moderate if one or more domains had “unclear risk” score; and high if one or more domains had “high risk” score. The quality of the included observational studies was assessed using the Newcastle–Ottawa Quality Assessment Scale [16]. This scale evaluates studies through the selection of study groups, the comparability of the groups, and the ascertainment of exposure (for case–control studies) or the assessment of outcomes (for cohort studies). The studies are scored from zero (the weakest study) to nine (the strongest study) according to Newcastle–Ottawa scale. The risk-of-bias assessment for each study included in the systematic review is summarized in complementary statement.

## Results

### Study selection

Overall, 2168 publications were initially identified in this review through doing search on PubMed (*n* = 1053), Web of Science (*n* = 59), Scopus (*n* = 767), Cochrane (*n* = 244), Embase (*n* = 45) and the reference lists of all relevant articles (*n* = 15). Considering title and abstracts of articles, 185 duplicate articles and 1935 unrelated articles were excluded. The full text of 45 publications were studied for further evaluation. Finally, 26 eligible studies (17 case–control, 6 trials, and 3 cohorts) were selected in the current systematic review (Fig. 1).

### Study quality assessment

Details of quality assessment of included studies are presented in Additional file 1: Tables S1, S2 and S3.

### Study characteristics

The characteristics of 26 selected studies in this systematic review are shown in Table 1. These studies have been published between 2004 and 2022. Five studies were exclusively performed on female subjects [17–22] and the others were done on both genders. The age of subjects in the selected studies was above 6 years old. In case–control studies 28 underweight, 683 overweight or obese and 628 normal weight subjects; in cross-sectional studies 311 subjects; in cohort studies 29,319 subjects and in interventional weight loss studies 295 subjects with overweight or obesity and 51 subjects with normal weight participated.

**Table 1:** Characterized of included studies

**Author (year)**	**Study design**	**Population**	**Age**	**Method of Taste assessment**
Jilani (2022) [23]	Cross-sectional	1938 children	7–11	**Taste threshold:** 5 watery solutions prepared with distilled water, with ascending concentrations of sucrose (8.8–46.7 mmol/l, sweet), sodium chloride (3.4–27.4 mmol/l, salty), monosodium glutamate (0.6–9.5 mmol/l, umami) or caffeine (0.26–1.3 mmol/l, bitter)/ using the paired comparison staircase method
Costanzo (2021) [24]	Cross-sectional	36 men and women	18–55	**Taste threshold:** twelve concentrations of oligofructose solutions determined using a validated ascending forced choice triangle methodology
Nishihara (2019) [17]	Intervention Parallel	Women: 27 obesity, overweight/ 24 normal weight	21–64	**Taste threshold:** Two-alternative, forced choice staircase procedure. Pairs of solutions, one of which was sucrose solution and the other deionized water. The concentration for the sucrose solution began at 1 × 10–4 M. to choose the one they thought contained the sweet taste and continued until the choice were correct based on especial criteria **Taste preference:** forced-choice, paired-comparison tracking technique (differed in the concentration of sucrose and ask to choose one preferred)
Vignini (2019) [25]	Case–control	30 normal-weight /19 overweight/22 obesity	> 32	**Taste sensitivity**: Filter paper strips / four different concentrations / self- assessment according to a multiple-choice question. Sucrose (.05- 0.4)/ sodium chloride (0.016- 0.25), citric acid (0.05- 0.3)/ quinine hydrochloride (0.0004- 0.006)
Mameli (2019) [26]	Case–control	children:34Obese /33 normal weight	6–14	**Taste sensitivity: ‘**Taste Strips’ method. Total number of 18 paper strips were used/ four different concentrations for each taste qualities (sweet, sour, salty and bitter) and two blank strips/self- assessment according to a multiple-choice question
Uygun (2019) [18]	Case–control	52obese/15 normal weight women	18–55	**Taste threshold:** sucrose concentrations (1.25 * 10^3^ to 6.4 * 10^1^ M)/ scale from 1 to 4
Proserpio (2018) [27]	Case–control	45obese/40 normal weight	18- 65	**Taste perception:** filter papers (Whatman) were soaked in a saturated aqueous PROP (6-n-propyl-2-thiouracil) / Comparing the average perceived bitterness of PROP papers with those of PROP solutions, PROP paper falls between the perceived bitterness of 0.001 and 0.0032 M PROP
Noel (2017) [28]	Cohort	93	young adults	**Taste intensity:** Three concentrations/ gLMS scale Sucrose (27.0, 81.0, and 243.0 mmol/L) / sodium chloride (33.3, 100.0, and 300.0 mmol/L)/ citric acid concentrations (1.0, 3.0, and 9.0 mmol/L)/ quinine concentrations (0.056, 0.168, and 0.498 mmol/L.)
Hardikar (2017) [29]	Case–control	23 obese (OB; BMI > 30), and 31 lean	18–35	**Taste threshold:** Using different concentrations/ adaptive Bayesian staircase procedure (QUEST) were continued until correct answer (sucrose, NaCl, citric acid, quinine)
Fernandez-Garcia (2017) [22]	Case–control	17 Low weight / 77 normal weight / 12 overweight/ 28 obesity/ 45 morbid obesity	18–65	**Taste sensitivity:** Taste strips/ The strips were placed on the left and right sides of the anterior third of the extended tongue/ Using different concentrations. 0.4- 0.05 g/ml sucrose/ 0.3- 0.05 g/ ml citric acid/ 0.25- 0.016 g/ml sodium chloride/ 0.006- 0.0004 g/ml quinine hydrochloride
Burgess (2016) [19]	Intervention (Low carbohydrate /low fat diet)	Women: 69 obese	44.2	**Taste threshold:** Strawberry milk varying in sucrose (0%, 15% and 30% wt/vol) / visual analogue scale **Taste preference:** salad dressing fat (10%, 30%, 50% wt/vol) / visual analogue scale
Newman (2016) [13]	Intervention	53 Overweight and obese	18–75	**Taste thresholds:** using triangle tests with ascending forced choice **Taste preference:** different foods including cream cheese, vanilla yogurt, chocolate mousse. / 9-point hedonic scale
Sauer (2016) [30]	Intervention	60 Obese/27 normal weight	9–17	**Taste perception:** Taste strip/ 4 different concentrations which have been conducted by filter paper / self- assessment according to a multiple-choice question 0.4—0.05 g/mL sucrose/ 0.3- 0.05 g/mL citric acid/ 0.25–0.016 g/mL sodium chloride/ 0.006–0.0004 g/mL quinine-hydrochloride **Subjective taste preferences:** asking if participants had a preferred taste
Proserpio (2016) [31]	Case–control	51 obese/ 52 normal weight	40.17 ± 10.79	**Taste sensitivity:** Seven concentrations of Sucrose, caffeine, sodium chloride, citric acid, and oleic acid were prepared in mineral water Sucrose (0.16- 40) / Sodium chloride (0.06 – 4)/ Caffeine (0.003 – 2) / Citric acid (0.33 – 50) / Oleic acid (0.02- 30) Taste **thresholds:** 3-AFC method
Ferna´ndez-Aranda (2016) [32]	Case–control	Women: 59 obese /36 normal weight	37.5	**Taste sensitivity:** Taste strips/ The strips were placed on the left and right sides of the anterior third of the extended tongue. Using different concentrations. 0.4- 0.05 g/ml sucrose/ 0.3- 0.05 g/ ml citric acid/ 0.25- 0.016 g/ml sodium chloride/ 0.006- 0.0004 g/ml quinine hydrochloride
Park (2015) [33]	Case–control	23 normal weight/ 18 overweight	20–29	**Taste threshold:** Electrogustometry (EGM) method were measured on both sides of the anterior and posterior tongue bases / 22 different thresholds, ranging from 3 uA (–8 dB) to 400 uA (34 dB), in a manner similar to pure-tone audiometry. /10 different concentrations of sodium chloride (0.016–0.9), sucrose (0.05–0.2), citric acid (0.05- 0.6), quinine hydrochloride (0.00001- 0.03) was administered
Skrandies (2015) [34]	Case–control	11 underweight/ 30 normal weight/ 18 overweight /7 obese	20–65	**Tats threshold:** Taste strip/ 4 different concentrations which have been conducted by filter paper / visual analogue scale
Bertoli (2014) [35]	Intervention	66 overweight/obese	> 65	**Taste threshold:** Three-alternative-forced-choice method. Five concentrations of sucrose, caffeine, sodium chloride and citric acid / 5 triads of samples marked with three-digit numbers
Ettinger (2012) [21]	Case–control	women:50 normal/ 21 overweight	18—49	**Taste thresholds:** Six concentrations of sucrose solutions (0.2%,—1.2% w/v) using the ascending forced-choice trial method
Overberg (2012) [12]	Case–control	99 obese/ 94 normal weight	6–18	**Taste sensitivity:** Taste strips made from filter paper were impregnated with four different concentrations (sweet: 0.4- 0.05 g/ml sucrose; sour: 0.3- 0.05 g/ml citric acid; salty: 0.25–0.016 g/ml Sodium Chloride; umami: 0.25- 0.016 g/ml monosodium glutamate; bitter: 0.006- 0.0004 g/ml quinine-hydrochloride) plus two blank strips / 5-point rating scale
Sartor (2011) [36]	Case–control	Normal weight 22/ overweight, obese 11	22.8 ± 2.5	**Taste sensitivity:** Eleven concentrations of sucrose (0, 0.5- 2.75 log[sucrose] mol/L) and seven concentrations of sodium chloride (1- 2.5 log [NaCl] mol/L)/ gLMS of intensity (150 mm)
Umabiki (2010) [20]	Intervention	Women: 20 overweight or obese	55	**Taste threshold:** 10 different concentrations (0.0098 – 50,000%) by forced-choice staircase method
Matsushit (2009) [37]	Cohort	29,103	middle-aged	**Taste preference:** Kotteri is a word that all Japanese would know, indexing a taste as common as sweet or sour, and described as a rich and heavy taste in Japanese dictionary. / self- assessment according to a multiple-choice question
Pasquet (2007) [38]	Case–control	39 obese/ 48 non-obese	11.5–18	**Taste threshold:** sucrose (2.0 to 1000 mM), fructose (2.0 to 1000 mM), citric acid (0.40 to 25 mM) and quinine hydrochloride (0.4400 mM), whereas the solutions of sodium chloride (1.77 to 1000 mM) and PROP (15 solutions: 0.0013.2 mM)/ visual analogue scale
Simchen (2006) [39]	Cross-sectional	311 men and women	< 65 > 65	**Taste sensitivity:** Four different concentrations of sodium chloride (3.2- 100 mmol/l), sucrose (0.0032,—0.1 mol/l), citric acid (0.63- 5 mmol/l) and quinine hydrochloride (3.8- 40 mmol/l)/ 0–100 scores by the FIZZ Software
Salbe (2004) [40]	Cohort	123	> 18	**Hedonic response:** Solutions of nonfat milk (0.1% fat), whole milk (3.5% fat), half and half (11.3% fat), and cream (37.5% fat) containing 0%, 5%, 10%, or 20% sugar by weight/ 100-mm visual analogue scale

*gLMS* General Labeled Magnitude Scale, *LMS* The labeled magnitude scale, *EGM* Electrogustometry, *3-AFC* 3 alternative forced choice

### Measurement scale

The taste strip scale including different concentrations of each component (sucrose, citric acid, sodium chloride, PROP (n-propylthiouracil) or quinine hydrochloride or caffeine) dissolved in tap water were used to measure taste sensitivity and threshold in all studies except two studies in which strawberry milk, salad dressing, sweetness and creaminess of milk were used to assess the sweet and fat taste status [19–22, 40] and determined using visual analogue scales or self- assessment according to a multiple-choice question. The sweet and fat taste preferences were assessed by different concentrations of sucrose, milk, salad dressing, cream cheese, vanilla, yogurt, chocolate mousse and determined using visual analogue scales or self- assessment according to a multiple-choice question [13, 17, 19].

### Overall taste sensitivity, taste threshold and taste preference among non- obese and obese individuals

#### Sweet taste sensitivity and threshold

Seventeen cross-sectional studies were included. Eight studies indicated that the perception and sensitivity of sweet taste was lower and threshold of sweet taste was higher in subjects with overweight/ obese than in normal-weight [12, 21–23, 25, 26, 31, 36, 39]. Three studies showed an inverse result in sweet taste (sensitivity, threshold) among different weight/BMI status [24, 29, 38]. No significant difference was seen in five studies [32–34, 39, 41] Table 2.

**Table 2:** Comparison taste perception between obese and non-obese

Author (year)	BMI bassline (kg/m2) / Z- score	Correlation	Taste Threshold				Taste sensitivity				
			**Sweet**	**Salt**	**Sour**	**Bitter**	**Sweet**		**Salt**	**Sour**	**Bitter**
Costanzo * (2021) [24]	0.5 (Z-score)	-	DS	DS	-	NS	-	-		-	-
Costanzo (2021) [24]	19–35	-	IS	-	-	-	-	-		-	-
Vignini (2019) [8, 25]	36. 9 ± 5. 7 obese 27. 9 ± 1.4 overweight	*r* = -0.36 Total	-	-	-	-	IS	IS		IS	IS
Mameli (2019) [26]	23.9 (> + 2SD)	-	-	-	-	-	IS	IS		IS	IS
Uygun (2019) [18]	≥ 30	-	-	-	-	-	IS	-		-	-
Proserpio (2018) [27]	37.57 ± 0.77	-	-	-	-	IS	-	-		-	-
Hardikar (2017) [29]	33.8	-	-	-	-	-	DS	DS		NS	NS
Fernandez-Garcia (2017) [22]	17.9 ± 0.51 low weight 21.6 ± 1.7 normal weight 26.8 ± 0.9 overweight 35.2 ± 2.6 obese 46.3 ± 5.3 morbid obese	*r* = -0.301 Sweet *r* = -0.388 Sour *r* = -0.237 Salt *r* = -0.239 Bitter *r* = -0.407 Total	-	-	-	-	IS	IS		IS	IS
Proserpio (2016) [31]	34.08 ± 4.29	-	DS	DS	DS	DS	-	-		-	-
Ferna´ndez-Aranda (2016) [32]	22.4 ± 2.6 normal weight 42.7 ± 6.6 obese	-	-	-	-	-	NS	NS		IS	NS
Park (2015) [33]	27.62 ± 2.57	-	NS	DS	NS	NS	-	-		-	-
Skrandies (2015) [34]	18.81 underweight 22.13 normal weight 27.32 overweight 34.38 obese	*r* = -0.21 Total *r* = -0.35 Salt *r* = 0 sweet, sour, bitter	NS	DS	NS	NS	-	-		-	-
Ettinger (2012) [21]	≥ 25	-	DS	-	-	-	-	-		-	-
Overberg (2012) [12]	> 97th percentile	*r* = -1.51 Total	-	-	-	-	IS	IS		IS	IS
Sartor (2011) [36]	24.7 ± 4.7	-	-	-	-	-	IS	IS		-	-
Pasquet (2007) [38]	39.59 ± 6.0 obese 21.09 ± 2.5 non- obese	*r = 0 Total	IS	IS	NS	NS	-	-		-	-
Simchen (2006) [39]	≥ 28	-	-	-	-	-	NS	NS		IS	IS

*DS* Direct Significant (taste variable was increased in increased wight status), *IS* Inverse Significant (taste variable was decreased in increased weight status), *NS* Non significant

^*^There were some correlations in both genders separately

^*^The study sample group is a sub-sample of one European cohort study

#### Salt taste sensitivity and threshold

Fourteen cross-sectional studies were included. Nine studies indicated that the sensitivity of salt taste was lower and threshold of salt taste was higher in subjects with overweight/ obese than in normal-weight subjects [12, 23, 25, 26, 31, 32, 34, 36]. Three studies showed sensitivity of salt taste was lower and threshold was higher in obese compared than non- obese individuals [29, 38, 41]. However, two studies showed no significant difference in perception (threshold and sensitivity) between obese and normal weight individuals [32, 39] Table 2.

#### Sour taste sensitivity and threshold

Twelve cross-sectional studies were included. Seven studies showed a significant difference in sensitivity and threshold of sour taste between non- obese and obese individuals [12, 22, 25, 26, 31, 32, 36, 39]. In one study, the threshold was higher in overweight/ obese than non-obese subjects [41]. Three studies showed no a significant difference in perception between non- obese and obese individuals [33, 34, 38] Table 2.

#### Bitter taste sensitivity and threshold

Fourteen cross-sectional studies were included. Seven studies showed the sensitivity of bitter taste was lower and the threshold of bitter taste was higher in overweight/obese individuals [12, 22, 25, 26, 31, 38, 39]. Six studies did not show any significant difference [23, 29, 32–34, 38, 41] Table 2.

### Overall taste sensitivity, threshold and preference after weight changes

#### Sweet taste sensitivity and threshold and sweet and fat preference

There were six studies investigating the sweet sensitivity and threshold [13, 17, 19, 20, 30, 35]. Although the sweet sensitivity and threshold improved after weight loss interventions in the most studies, it was just significantly changed in one study [20]. One study showed deterioration the perception of sweet taste [30] Tables 3 and 4.

**Table 3:** Outcomes of longitudinal studies

**Author (year)**	**Weight change**	**BMI Bassline (kg/m2)**	**Duration**	**Taste perception**				**Preference**
				**Sweet**	**Salt**	**Sour**	**Sweet**	**Fat**
Noel (2017) [28]	+ 3.9%	21.9	8 months	↓ in male	↓ In male	↑ in female	-	-
Matsushit (2009) [37]	≥ + 5 kg	> + 23	10 years	-	-	-	↑	-
Salbe (2004) [40]	+ 9.0 ± 10.8 kg men + 8.9 ± 9.0 kg women	-	5.5 ± 3.0 years	-	-	-	↑	↑

**Table 4:** Outcomes of interventional studies

Author (year)	BMI Bassline (kg/m2) or z score	Weight change	Duration	Taste threshold					Taste sensitivity				Preference	
				**Sweet**	**Salt**	**Sour**	**Bitter**	**Fat**	**Sweet**	**Salt**	**Sour**	**Bitter**	**Sweet**	**Fat**
Nishihara (2019) [17]	29.8 ± 0.5	–10.7 kg	30 weeks	↔	-	-	-	-	-	-	-	-	↓	-
Newman (2016) [13]														
Low fat diet Low calorie diet	32.3 ± 0.7	-2.5 ± 0.4 kg	6 weeks	↔	↔	-	-	↓	-	-	-	-	-	↔
		-2.2 ± 0.4 kg	6 weeks	↔	↔	-	-	↓	-	-	-	-	-	↔
Burgess (2016) [19]														
(Low fat/ low carbohydrate diet)	34.4	-7.0 ± 0.8 kg	3 months	↔	-	-	-	↔	-	-	-	-	↔	↓
		-9.1 ± 0.9 kg	6 months	↔	-	-	-	↔	-	-	-	-	↓	↓
Sauer (2016) [30]	2.51 ± 0.6 (Z-score)	-4.3 ± 2.5 kg	26.4 ± 8.2 days	-	-	-	-	-	↓	↔	↑	↔	-	-
Bertoli (2014) [35]	27.9 ± 1.6 overweight 34.8 ± 4.6 obese	-2.5 ± 1.7% < 5% weight loss	3 months	↔	↔	↔	↔		-	-	-	-	-	-
		-7.8 ± 2.5% ≥ 5% weight loss	3 months	↔	↔	↔	↔		-	-	-	-	-	-
Umabiki (2010) [20]	26.1 ± 1.7	-3.6 ± 2.0 kg	12 weeks	↓	-	-	-	-	-	-	-	-	-	-

The Fat threshold or sweet and fat preference were decreased in three weight loss interventional studies [13, 17, 19]. In addition, the sweet and fat preference was increased along with weight gain in two large longitudinal cohort studies [37, 40]. The sweet and salt taste perception was decreased along with weight gain in males in one cohort study [28].

#### The sensitivity and threshold of salt taste

The sensitivity and threshold of salt taste did not change after weight loss interventions in three studies [13, 30, 35] Tables 3 and 4.

#### The sensitivity and threshold of sour and bitter tastes

There were two studies investigated the sensitivity and threshold of sour and bitter [30, 35]. One study found that sour sensitivity was increased after weight loss interventions [30] Tables 3 and 4.

## Discussion

Food choice can be affected by various factors including social factors such as family and cultural norms; smoking, physical factors such as access, availability, and time; economic factors such as cost and income; individual psychological factors such as stress, mood and attitudes; and biological factors such as appetite and taste as well as some medications [42, 43]. It is indicated that taste preference and hedonics can impact on food choice and lead to intake the high calorie foods and consequently result in obesity. On the other hand, obesity can affect taste perception and brain reward response as taste preference and may result in increased food intake and further weight gain and obesity [44]. Consistent with these findings, there are many observational studies reported an inverse relationship between taste perception of sweet, salt, sour, and bitter and weight status in adults and children [12, 21, 22, 25–27, 31, 36]. However, some studies reported diverse findings. Harakiri et al. reported no significant difference in sour and bitter tastes and also high sensitivity of sweet and salt tastes in obese compared to the non-obese individuals [29]. Two studies showed threshold (one study for salt and sour and another for sweet and salt) was lower in obese compared to non-obese individuals [38, 41]. However, in both of studies non-obese included normal weight and overweight (BMI < 30). So, the outcomes may have been affected by this category. In contrast, two studies compared taste function among subjects with different categories of BMI [22, 34]. Garcia et al. showed although the sensitivity of sweet, salty, sour and bitter was lower in obese than the normal weight, a tendency to decline was shown in groups with lower (BMI < 18.5) and higher (BMI > 30) BMI [22]. In addition, Skrandies et al. observed only the threshold of salt was significantly higher in BMI > 25 compared to other BMI categories [34]. Additionally, the outcomes of other studies found taste function was lower in overweight or obese than the normal weight. Park et al. found high thresholds of all tastes in obese subjects but just the threshold of salty had significant difference [33]. Moreover, Simchen et al. showed that the sensitivity and perception of sour and bitter tastes were age-dependent and BMI-dependent in overweight subjects with BMI 28 < was lower than BMI 28 > and aged < 65 years old was higher than subjects aged ≥ 65 years old [39].

Furthermore, there are few longitudinal studies reported a linear correlation between taste preference of sweet or fat and weight gain [37, 40]. Some experimental animal and neuroimaging human studies confirmed the reward generation and taste responsiveness was lower in obese subjects compared with normal weight subjects. Animal studies found that obese rats fed in a high-fat diet had a higher sucrose and fat preference compared with lean controls [45–47], which it may increase the prevalence of obesity. The most observational studies used basically similar scale to assess the sensitivity, threshold and perception, whatever, there had differences such as the number of dilution steps, modes of stimulation (whole mouths against localized stimulation), differences in threshold algorithms (ascending against adaptive methods), tasks (2- or 4- alternative forced choice; AFC), as well as the type of concentration scale (linear against log-linear).

Although several observational and longitudinal studies reported an inverse correlation between taste sensitivity and preference in overweight or obese subjects, there is still unclear that weight loss may reverse taste perception and decrease taste preference in overweight or obese subjects. Recent studies indicated the effects of weight loss surgery on decreasing the preference of sweet and fat taste in obese subjects [48–50]. As it was unclear that weight loss may improve taste preference and perception, some studies with nonsurgical weight loss trials have been done. In Nishiharas study, although the preference and palatability of sweet was decreased during weight loss intervention, the perception of sweet taste did not change significantly [17]. Newman et al. showed that both low fat (25%) and low-calorie diet-induced weight loss improved the perception of fat, sweet and salt taste, and decrease fat preference but only the change of fat perception was significant [13]. In one trial study, only the perception of sour taste was increased after the weight loss of 4.3 kg in children with obesity, however, the threshold of salt and bitter taste did not change and the threshold of sweet taste was reduced. So, the authors suggested that the reduction of the perception of sweet taste may be a physiological response against dietary weight loss in children [30]. Bertoli study showed none of the four-taste perception did change in subjects with obesity after 3 months of intervention led to either < 5% or ≥ 5% weight loss [35].

It is suggested that having various study designs such as duration of intervention, may be a reason that the results of studies were not consistent. Moreover, in some studies the variables like single nucleotide polymorphisms (SNP) were not considered. In Burgess et al. study, the preference of sweet and fat taste was decreased after six months of a weight loss diet, while did not reduce after 3 months [19]. It was indicated that as in most interventional studies the main aim was weight loss, the subjects did not achieve normal or steady weight at the end of study.

Recently, several studies suggested a link between hormone leptin and the perception and preference of taste [17, 20, 51, 52]. These studies indicated that leptin can act as a modulator of peripheral taste receptors [52]. Nishihara and Burgess found that the preference and palatability of sweet and fat tastes was normalized in overweight or obese women after a weight loss intervention with losing 10.7 kg and 7 kg body weight respectively. So, Nishihara et al. suggested the role of leptin levels in altering the preference of sweet taste in obese women [17, 19]. Moreover, Umbaki et al. study found that the threshold of sweet taste was decreased in females with obesity after losing 3.6 kg body weight and suggested that this improvement was associated with serum leptin levels [20]. However, it is not clear why the taste preference is different in the subjects with obesity even in the same condition. It is indicated that although all neural centers and neurotransmitters (specifically dopamine) in the brain can control the palatability of foods, this neural network is influenced by genetic and weight status. The genetic taste blindness to the bitter taste of PROP predicts the increased preferences of sweet or fatty foods [53, 54]. Furthermore, some studies suggested the effects of gender on preference and perception of taste [12, 38]. Viginini found that the sensitivity and perception of taste was higher in females than males [25]. In one study in which the college students participated, the perception of sweet and salty was decreased in males after 3 months intervention led to 3.9 kg weight gain, while the perception of the sour taste was decreased in females without any change in perception of sweet and salt taste [28]. Another study also found that obese men had lower sensitivity as well as fungiform papillae number compared with women with obesity [27]. Sartor showed that young obese men had more desire for sweet taste than obese women [36]. It seems that the taste is highly affected by obesity in men compared with women with the same age.

As several factors can impact on food choice, we did not include studies in which a food questionnaire was used to assess the perception and preference of taste. However, most studies in which these scales were used showed that the subjects with obesity had higher preference of sweet and fat taste [55–57]. Lanfer et al. assessed the preference of taste in children in eight European countries and reported that obese children had higher preferences of fat and sweet considering the confounding variables [56]. Moreover, Lampuré et al. in a French cohort study found that higher fat preference predicts the obesity risk [55].

## Conclusion

Overall, as the results of all included articles in this review can be inferred the sensitivities or perceptions of four main tastes specifically sweet and saltary taste are lower in overweight and obese than normal weight subjects. Additionally, according to longitudinal studies, the preference of sweet and fat is increased along with weight gain in adults. Although the number of studies in this issue is insufficient, it seems that weight gain and obesity can lead to reduction of taste perception and increase the preference of sweet and fat. In addition, many both observational and interventional studies demonstrate taste function is more affected in men than women. It is suggested that the results of the available interventional studies are not conclusive and need further studies with the same and standard design adjusting cofounding variables including genetic, gender, age and food condition of subjects.

## Supplementary Information

**Additional file 1: Table S1.** Quality score of case-control studies. **Table S2.** Quality score of cohort studies. **Table S3.** Quality score of interventional studies.

## Data Availability

The datasets analyzed in this study are accessible by the corresponding author on any reasonable request. The dataset supporting the conclusions of this article is included within the article and its additional file.
