# Supplementary material for: Effects of weight change on taste function; a systematic review
Source: Nutr J. 2023 May 8;22:22. doi: 10.1186/s12937-023-00850-z (PMC10165840; doi:10.1186/s12937-023-00850-z)
Supplement: Supplementary file 1 — Additional file 1: Table S1. Quality score of case-control studies. Table S2. Quality score of cohort studies. Table S3. Quality score of interventional studies. [file 12937_2023_850_MOESM1_ESM.docx]

**Table S1. Case-Control Study Quality Score According to NEW CASTEL Risk of Bias Criteria**

| Study Author/Date | Selection, no. of stars (possible range 0 to 4 | Comparability, no. of stars (possible range 0 to 2) | Exposure, no. of stars (possible range 0 to 3) | Total, no. of stars (possible range 0 to 9) |
| --- | --- | --- | --- | --- |
| Jilani (2022) | ++ | ++ | ++ | High risk |
| Costanzo (2021) | ++ | ++ | ++ | High risk |
| Vignini 2019 | +++ | ++ | ++ | Low risk |
| Mameli 2019 | ++++ | ++ | +++ | Low risk |
| Uygun 2019 | ++ | ++ | ++ | High risk |
| Proserpio 2018 | +++ | ++ | ++ | Low risk |
| Hardikar 2017 | ++ | ++ | ++ | High risk |
| Fernandez-Garcia (2017) | ++++ | ++ | ++ | Low risk |
| Proserpio 2016 | ++++ | ++ | + | Low risk |
| Ferna´ndez-Aranda (2016) | ++++ | ++ | + | Low risk |
| Park 2015 | ++++ | ++ | ++ | Low risk |
| Lorenzo (2015) | ++++ | ++ | ++ | Low risk |
| Skrandies (2015) | ++++ | ++ | ++ | Low risk |
| Ettinger (2012) | ++++ | ++ | ++ | Low risk |
| Overberg 2012 | ++++ | ++ | ++ | Low risk |
| Sartor 2011 | +++ | ++ | ++ | Low risk |
| Pasquet (2007) | ++++ | ++ | ++ | Low risk |
| Simchen 2006 | ++ | ++ | ++ | High risk |

The Newcastle-Ottawa Scale is a tool designed to assess the quality of nonrandomized studies. Basically, a “star system” is used to evaluate a study by the selection of the study groups, the comparability of the groups, and the ascertainment of the exposure (for case -control studies) or the outcomes (for cohort studies). The higher the number of stars, the better the quality of the study.

The quality assessment of selection includes four items, each can be awarded a maximum of 1 star: (1) is the case definition adequate; (2) representativeness of the cases; (3) selection of controls; (4) definition of controls.
The quality assessment of comparability includes one item that can be awarded a maximum of 2 stars: comparability of cases and controls on the basis of the design or analysis.
The quality assessment of exposure includes three items that each can be awarded a maximum of 1 star: (1) ascertainment of exposure; (2) same method of ascertainment for cases and controls; (3) non-response rate. 7-9=Low risk, 4-6= high risk, 0-3= very high risk of bias.

**Table S2. Cohort Study Quality Score According to NEW CASTEL Risk of Bias Criteria**

|  | Selection  0-4 | Comparability  0-2 | Outcome  0-3 |
| --- | --- | --- | --- |
| Noel (2017) | +++ | ? | Very high risk |
| Matsushit (2009) | +++ | ++ | Very high risk |
| Salbe (2004) | +++ | ++ | Very high risk |

The Newcastle-Ottawa Scale is a tool designed to assess the quality of nonrandomized studies. Basically, a “star system” is used to evaluate a study by the selection of the study groups, the comparability of the groups, and the ascertainment of the exposure (for case -control studies) or the outcomes (for cohort studies). The higher the number of stars, the better the quality of the study.

The quality assessment of selection includes four items, each can be awarded a maximum of 1 star: (1) is the case definition adequate; (2) representativeness of the cases; (3) selection of controls; (4) definition of controls.
The quality assessment of comparability includes one item that can be awarded a maximum of 2 stars: comparability of cases and controls on the basis of the design or analysis.
The quality assessment of exposure includes three items that each can be awarded a maximum of 1 star: (1) ascertainment of exposure; (2) same method of ascertainment for cases and controls; (3) non-response rate. 7-9=Low risk, 4-6= high risk, 0-3= very high risk of bias

**Table S3. Study Quality Score According to Cochrane Risk of Bias Criteria**

| Study Author/Date | Risk of inadequate generation of a randomized sequence | Risk of inadequate concealment of allocation prior to assignment | Knowledge of allocated intervention by participants and personnel during the study | Knowledge of allocated intervention by outcome assessors | Inadequate assessment of incomplete outcome data | Suggestion of selective outcome reporting |
| --- | --- | --- | --- | --- | --- | --- |
| Nishihara 2019 | low | Unclear | Unclear | High | Unclear | low |
| Burgess 2016 | Low | Unclear | High | High | Unclear | Low |
| Newman 2016 | Low | Unclear | High | High | High | Low |
| sauer 2016 | Low | Unclear | High | High | Unclear | Low |
| Bertoli 2014 | Unclear | High | High | High | Unclear | Low |
| Umabiki 2010 | Unclear | High | High | High | Unclear | Low |

Low quality ranges: −6 to 0, medium quality ranges: 1-3, high quality ranges: 4-6
